# Frozen parental care behavior in a pseudoscorpion trapped in Eocene Baltic amber

**DOI:** 10.1016/j.isci.2026.117264

**Published:** 2026-08-21

**Authors:** Chen Feng, Corentin Jouault, Qingqing Zhang, Arnoud Boom, Jana Christophoryová, Xiangbo Guo, Yuhe Gao, Chunpeng Xu, Yu Liu

**Affiliations:** 1 Yunnan Key Laboratory for Palaeontology, Institute of Palaeontology, Yunnan University, Kunming 650500, China; 2 MEC International Joint Laboratory for Palaeobiology and Palaeoenvironment, Yunnan University, Kunming 650500, China; 3 State Key Laboratory of Palaeobiology and Stratigraphy, Nanjing Institute of Geology and Palaeontology, Chinese Academy of Sciences, Nanjing 210008, China; 4 School of Geography, Geology, and the Environment, University of Leicester, University Road, Leicester LE1 7RH, UK; 5 Oxford University Museum of Natural History, University of Oxford, Parks Road, Oxford OX1 3PW, UK; 6 Department of Zoology, Faculty of Natural Sciences, Comenius University, Mlynská Dolina, Ilkovičova 6, SK 842 15 Bratislava, Slovakia; 7 Key Laboratory of Zoological Systematics and Application, College of Life Sciences, Hebei University, Baoding 071002, China; 8 Publicity Center of Beijing Forestry and Parks, Beijing 101118, China; 9 Division of Terrestrial Zoology, Senckenberg Natural History Museum, 60325 Frankfurt, Germany; 10 Institut für Zoologie und Evolutionsforschung, Friedrich-Schiller-Universität Jena, 07743 Jena, Germany

**Keywords:** pseudoscorpiones, eocene fossil, phoresy, brood sac

## Abstract

Direct fossil evidence of behavior is rare, which limits our understanding of the early evolution of social and reproductive strategies. Parental care and phoresy are two important life-history traits in arthropods: parental care can improve offspring survival, whereas phoresy enables small animals to disperse by attaching to larger, more mobile carriers. Pseudoscorpions are a diverse and ecologically widespread group of arachnids, with parental care well documented among living species. However, the fossil record of this behavior remains extremely limited. Here, we report the earliest known evidence for the combined occurrence of parental care and phoresy in pseudoscorpions, preserved in approximately 35-million-year-old Baltic amber. The specimen preserves a female pseudoscorpion carrying a brood sac while attached to a dipteran, apparently grasping one of the fly’s hind legs with her left pedipalp. This association shows that brood care and phoretic dispersal were already coupled by the Eocene. It further suggests that phoresy may have allowed brooding pseudoscorpions to disperse while maintaining physical protection of their developing offspring.

## Highlights

• Earliest fossil evidence of simultaneous parental care and phoresy in pseudoscorpions
• A brooding pseudoscorpion appears to grasp a fly leg in Baltic amber
• Parental care and phoretic dispersal were coupled by the Eocene

## Graphical abstract

**Figure:**
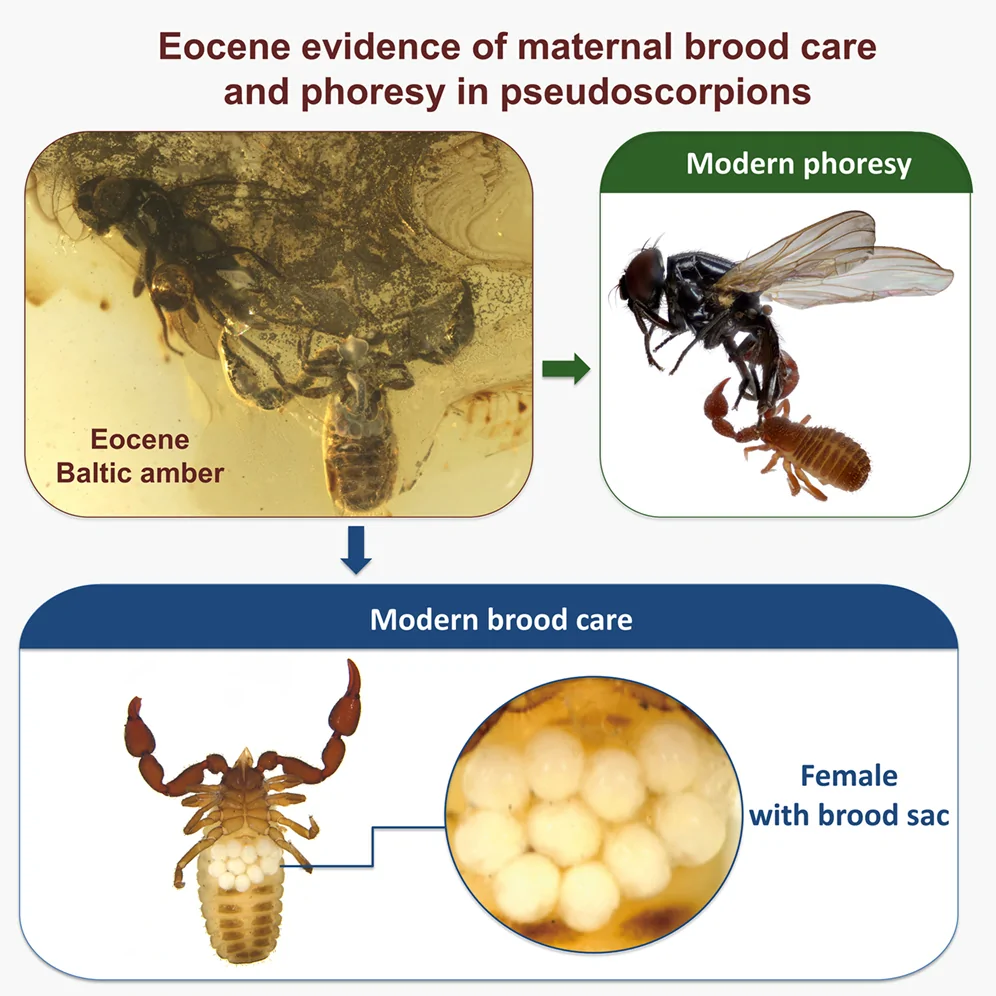

## Teaser

Biological sciences; Zoology; Paleobiology

## Introduction

Pseudoscorpions are small arachnids that superficially resemble scorpions but lack a metasoma and stinger. They are a diverse and ecologically widespread arachnid group in which parental care occurs, with more than 4,300 living species.^1^^,^^2^ They are globally distributed and usually occupy cryptic terrestrial microhabitats, including leaf litter, soil, spaces beneath bark, and the undersides of rocks, where they prey on small arthropods.^1^ The fossil record of pseudoscorpions extends to the Devonian, approximately 390 million years ago (Ma),^3^^,^^4^^,^^5^ but the group is conspicuously rare in most other Paleozoic deposits. This scarcity probably reflects a strong taphonomic bias: many Paleozoic Konservat-Lagerstätten pseudoscorpions were deposited in lacustrine or otherwise aquatic settings, environments that pseudoscorpions did not inhabit and in which their preservation would have been unlikely.^6^^,^^7^ Consequently, much of their early evolutionary history remains difficult to reconstruct, and the timing of key behavioral innovations, including social and reproductive behaviors, remains particularly obscure.

The fossil record becomes more informative in the Mesozoic, probably owing to the widespread production and preservation of fossiliferous resins during the cretaceous resinous interval, approximately 125–72 Ma.^7^^,^^8^^,^^9^ Even so, direct evidence of reproductive behavior in fossil pseudoscorpions is exceptionally rare. Phoresy, a dispersal strategy in which one organism attaches to another for transport, is relatively well documented in Baltic amber pseudoscorpions.^10^^,^^11^^,^^12^ Fossil evidence of egg-carrying or brood-carrying females is considerably rarer, with isolated examples reported from Baltic amber^13^^,^^14^^,^^15^^,^^16^ and younger deposits such as Mexican or Dominican amber.^10^^,^^17^ However, the co-occurrence of brood care and phoretic attachment in a single fossil pseudoscorpion has not been documented previously.

**Figure 1:**
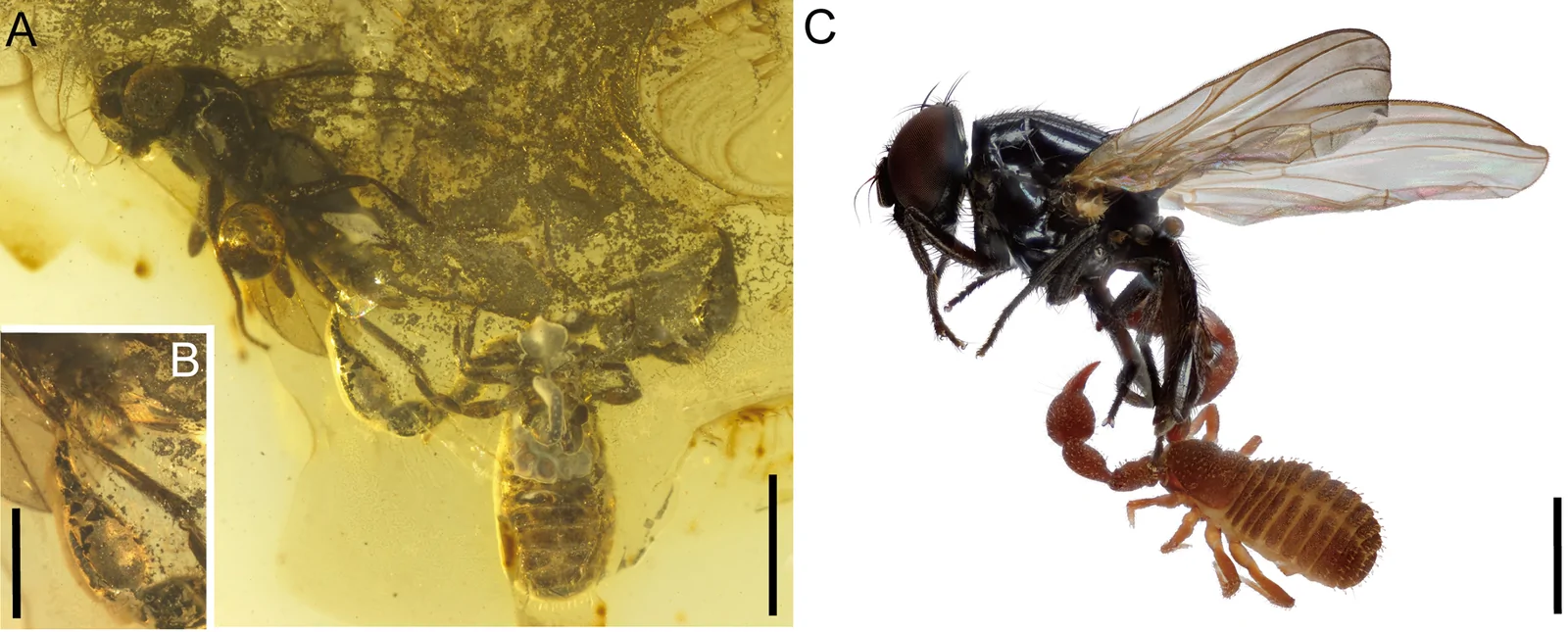
Association of an Iocheiratan pseudoscorpion with a dipteran host in Baltic amber (A) Ventral view of the Iocheiratan female and the dipteran host. (B) Pedipalp of the pseudoscorpion in contact with the leg of the dipteran. (C) Extant example of *Chernes* sp. (Chernetidae) being phoretic on a dipteran host. Scale bars, 1 mm (A and B) and 0.5 mm (C).

In extant arthropods, parental care is generally associated with increased offspring survival under environmental pressures such as predation and desiccation.^18^^,^^19^^,^^20^ In pseudoscorpions, phoretic dispersal by brood-carrying females may also have facilitated the colonization of new microhabitats while maintaining protection of developing offspring. Although the functional significance of this behavioral association cannot be tested directly in fossil material, the specimen described here provides the earliest direct evidence that brood care and phoretic behavior could occur simultaneously in pseudoscorpions. This finding offers new insight into the evolutionary interplay between reproductive behavior and dispersal strategies in this arachnid lineage (Figures 1A and 1B).

## Results

In the amber specimen, the left pedipalp of a female pseudoscorpion is positioned next to, and appears to grasp, one of the hind legs of the associated dipteran (Figures 1A and 1B). The dipteran can be assigned to Acalyptratae (Brachycera: Diptera), based on its preserved morphology (Figure 1A). This phoretic behavior is strikingly similar to that observed in extant pseudoscorpions, which commonly attach to insects for dispersal (Figure 1C). An opaque cloud of bubbles obscures the dorsal region of the female pseudoscorpion’s body (Figures S1A and S1B). Nevertheless, optical observations support assignment of the specimen to Iocheirata on the basis of several apomorphic features: the trichobothrial pattern of the chela, with trichobothria ib and isb situated on the lateral face; the fusion of metatarsi and tarsi in all legs; and the presence of venom glands in at least one chelal finger (see also Supplementary Text and Figures S1C and S1D).^21^ The absence of dentate subterminal tarsal setae further suggests a tentative placement within Panctenata. This assignment remains provisional because several key diagnostic characters, including the presence or absence of a velum and lamina and the condition of the serrula exterior, cannot be assessed in the specimen.^21^

**Figure 2:**
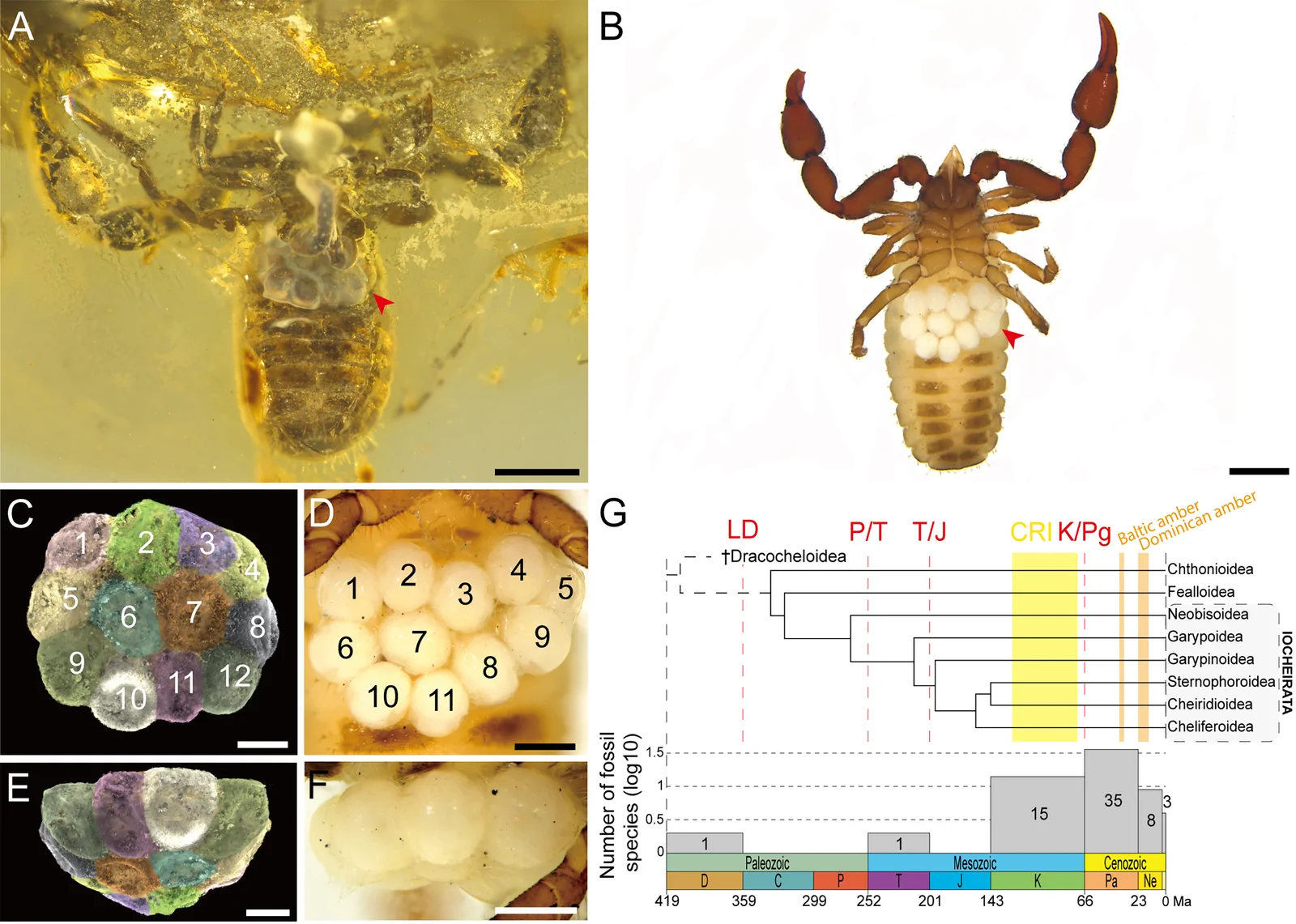
Evidence of parental care in an Iocheiratan female preserved in Eocene Baltic amber (A) Ventral view of the Iocheiratan female preserved in Baltic amber, carrying a brood sac (red arrow). (B) Ventral view of an extant specimen of *Chernes* sp. (Chernetidae), carrying a brood sac (red arrow). (C) 3D rendering of the amber piece showing the brood sac consisting of 12 eggs (frontal view). (D) An extant specimen of *Chernes* sp. (Chernetidae) showing the brood sac consisting of 11 eggs. (E) Lateral view of the 3D reconstruction. (F) Lateral view of extant specimen. (G) Phylogenetic placement of Iocheirata within Pseudoscorpiones, based on topology and divergence time (for extant lineages) from Benavides et al.,^21^ alongside the temporal positioning of Baltic and Dominican ambers, major mass extinction events (LD, Late Devonian; P/T, Permo-Triassic; T/J, Triassic-Jurassic; K/Pg, Cretaceous-Paleogene), and the cretaceous resinous interval (CRI). The graph also illustrates pseudoscorpion species diversity through geological time (data from World Pseudoscorpion Catalog). Scale bars, 0.5 mm (A–F).

Notably, the specimen also preserves a brood sac attached to the female, providing rare evidence of reproductive behavior in fossil pseudoscorpions. Optical observations reveal the brooding female in both the fossil specimen and an extant analogue, showing comparable ventral configurations (Figures 2A and 2B). Using micro-computed tomography (μCT) and wide-field fluorescence microscopy, we resolved detailed morphological features of the brooding female and its brood sac (Figures 2C–2E). Mineral inclusions within the amber, especially near the dipteran and the pseudoscorpion’s chelicerae, prevent complete three-dimensional reconstruction of the body. These artifacts do not, however, compromise reconstruction of the brood sac itself (Figure 2C). The resulting model reveals 12 eggs enclosed within a nearly elliptical sac. Individual eggs measure approximately 50–80 μm in diameter and are arranged in two layers: an outer layer of larger, more clearly defined eggs and an inner layer that is less distinct because of overlap. All eggs show some degree of collapse, most likely resulting from taphonomic alteration. The number and size of the eggs are comparable with those observed in extant pseudoscorpions such as *Chernes* sp. (Chernetidae) (Figures 2B, 2D, and 2F), confirming the interpretation of the structure as a genuine brood sac rather than an incidental inclusion or preservational artifact. Together, these observations provide rare direct evidence of both dispersal and reproductive behavior. Placing this finding in an evolutionary context, the systematic position of the specimen is shown in Figure 2G (light grey-shaded area).

## Discussion

In extant pseudoscorpions, carrying a brood-sac enhances offspring survival but may substantially restrict maternal mobility. This trade-off makes the association between the brooding female and a dipteran especially noteworthy. In the amber specimen, the female’s left pedipalp is positioned next to, and appears to grasp, one of the dipteran hind legs (Figure 1B). Although such close associations could in principle be interpreted as predation or failed predation, comparable pseudoscorpion-arthropod associations are generally better explained as transport, and the accidental-predation hypothesis has been rejected for modern examples.^22^^,^^23^ Predation in pseudoscorpions typically involves immobilization and feeding, whereas phoresy entails non-lethal attachment to a larger, more mobile host carrier for transport.^23^ The absence of evidence for prey consumption, together with the position of the pedipalp on the dipteran leg, therefore supports interpretation of the association as phoretic attachment.

Dipterans belonging to the relevant groups are commonly associated with soil, decaying organic matter, leaf litter, compost, or wood galleries, habitats that overlap strongly with the cryptic microhabitats occupied by pseudoscorpions (Figure 1C).^24^^,^^25^ This ecological overlap strengthens the interpretation that the association records a meaningful biological interaction rather than accidental co-entrapment in resin. The specimen therefore provides evidence that maternal brood care and phoretic dispersal could occur simultaneously in pseudoscorpions. Carrying a brood-sac would have protected the developing offspring and helped maintain a stable microenvironment during embryogenesis, whereas phoresy may have facilitated passive transport to new or ephemeral habitats, potentially offsetting the otherwise limited dispersal capacity of these small arachnids.^25^^,^^26^^,^^27^

The combination of these behaviors may have been particularly advantageous under ecologically challenging conditions. Developing embryos are vulnerable to predation, desiccation, hypoxia, and other environmental stresses, and parental care can reduce these risks by maintaining close physical protection of the brood.^19^ At the same time, brooding females likely experience reduced mobility and increased energetic costs. Phoresy may have mitigated these constraints by enabling dispersal without interrupting maternal care. Thus, the Baltic amber specimen suggests that parental investment and dispersal were not necessarily independent behavioral traits in Eocene pseudoscorpions, but could occur as complementary components of a broader reproductive strategy. This behavioral association may have contributed to the ecological persistence of pseudoscorpions in patchily distributed terrestrial microhabitats.

Our discovery of a well-preserved brooding female pseudoscorpion attached to a dipteran in Eocene Baltic amber provides the earliest direct fossil evidence for the co-occurrence of parental care and phoresy in pseudoscorpions. This finding extends the minimum age of this behavioral association to approximately 35 Ma and indicates that the coupling of reproductive investment with passive dispersal was already established by the Eocene, and possibly earlier (Figures 1A–1C). Given the rarity of direct behavioral preservation in the pseudoscorpion fossil record, the specimen offers an unusual and informative glimpse into the early evolution of reproductive and dispersal behaviors in this arachnid lineage.

### Limitations of the study

This study is based on a single fossil specimen and therefore does not allow quantitative assessment of the prevalence of parental care in extinct pseudoscorpions. Because fossilization captures only a single moment in time, the complete sequence and frequency of maternal behaviors cannot be directly observed. Nevertheless, the exceptional preservation of the adult female with associated offspring provides strong evidence for parental care. Future discoveries of additional fossil specimens from different geological deposits will be essential for evaluating the evolutionary distribution and broader evolutionary significance of parental care in pseudoscorpions.

## Resource availability

### Lead contact

Further information and requests for resources should be directed to and will be fulfilled by the lead contact, Yu Liu (yu.liu@ynu.edu.cn).

### Materials availability

The fossil specimen (YKLP-AMB-016; Figure 1A) has been deposited at the Yunnan Key Laboratory for Palaeobiology.

### Data and code availability

• All CT data reported in this paper have been deposited at Mendeley Data: 10.17632/xfc7zrcvgw.2 and are publicly available.
• This paper does not report original code.
• Any additional information required to reanalyze the data reported in this paper is available from the lead contact upon request.

## Acknowledgments

This research was funded by the Yunnan Revitalization Talent Support Program (to Y.L.). Y.L. and C.F. are currently visiting the 10.13039/501100000738University of Leicester (UoL), UK, supported by the 10.13039/501100004543China Scholarship Council and generously hosted by Professors Mark Williams and Arnoud Boom. Y.L. is grateful to the 10.13039/501100000738University of Leicester for awarding him an Honorary Visiting Professorship. J.C. received research support from the Scientific Grant Agency of the Ministry of Education of the Slovak Republic (grant no. 10.13039/501100006109VEGA 1/0817/25). C.X. is supported by the 10.13039/100005156Alexander von Humboldt-Stiftung as a research fellow and the Strategic Priority Research Program (B) of the 10.13039/501100002367Chinese Academy of Sciences (XDB0850000). Q.Z. is supported by 10.13039/501100001809National Natural Science Foundation of China (42562003) and Yunnan Fundamental Research (grant no. 202401CF070193). The authors also thank Dr. Alexssandro Camargo for identifying the dipteran host to the group Acalyptratae.

## Author contributions

Conceptualization, C.F., C.J., and Y.L.; methodology, C.F., X.G., J.C., and Q.Z.; investigation, C.F. and C.J.; writing – original draft, C.F., C.J., and Y.L.; writing – review and editing, C.F., C.J., A.B., C.X., and Y.L.; funding acquisition, Y.G. and Y.L.; supervision, A.B. and Y.L. All authors were involved in the analyses of the data and confirmed the manuscript.

## Declaration of interests

The authors declare no competing interests.

## STAR★Methods

### Key resources table

**Table:** 

REAGENT or RESOURCE	SOURCE	IDENTIFIER
**Biological samples**		

Fossil specimen	Yunnan Key Laboratory for Palaeobiology, Yunnan University	YKLP-AMB-016

**Software and algorithms**		

Drishti software (Version 3.2)	Limaye^28^	N/A
CorelDRAW *X*8	Corel Corporation	N/A
Adobe Illustrator CC2019	Adobe Systems Incorporated	N/A

**Deposited data**		

CT dataset	Mendeley Data	Mendeley Data: 10.17632/xfc7zrcvgw.2

### Experimental model and study participant details

#### Fossil specimen

The specimen is preserved in a clear yellow piece of Baltic amber with a dipteran syninclusion. The amber derives from the Blue Earth Formation, also known as the Blue Earth Member of the Prussian Formation, in the Russian exclave of Kaliningrad (54°41′19.5″N, 20°20′50.6″E). Palynological evidence dates this unit to the late Bartonian–Priabonian of the late Eocene, approximately 34–38 Ma.^4^^,^^29^^,^^30^ Additional information on the age and paleobiota of Baltic amber can be found in previous studies.^11^^,^^30^^,^^31^ The specimen is deposited in the Yunnan Key Laboratory for Palaeobiology, Institute of Palaeontology, Yunnan University, Kunming, China, under collection number YKLP-AMB-016. The amber piece measures 0.8 cm × 0.6 cm × 0.2 cm.

### Method details

#### Imaging

The amber piece was examined using a Keyence VHX-6000 3D microscope and a Zeiss Stemi 508 microscope for measurement and high-resolution photography, including non-reflective imaging, depth-composited imaging, and image stitching. Wide-field fluorescence images were acquired with a Leica THUNDER Imager DMi8, mainly using the 747 nm excitation line. Line drawings and explanatory figures were prepared in CorelDRAW *X*8 and Adobe Illustrator CC2019.

#### CT scanning and reconstruction

To generate a three-dimensional model of the specimen, we scanned it with a Zeiss Xradia 520 Versa X-ray microscope (Carl Zeiss X-ray Microscopy, Inc., Pleasanton, CA, USA). Scan parameters were 60 kV and 5 W, with an exposure time of 5 s per projection. The final voxel size was 0.8 μm. Data were collected over a rotation range of −92° to +92°, with 1,601 projection images acquired per segment. Reconstruction yielded 968 slices, which are publicly available in Mendeley Data (10.17632/xfc7zrcvgw.2). Reconstructed slices were exported as TIFF stacks and processed in Drishti v.3.2 (Limaye, 2012), in which screenshots of the three-dimensional models were also generated.
