# Supplementary material for: Frozen parental care behavior in a pseudoscorpion trapped in Eocene Baltic amber
Source: iScience. 2026 Aug 21;29(9):117264. doi: 10.1016/j.isci.2026.117264 (PMC13524697; doi:10.1016/j.isci.2026.117264)
Supplement: Supplementary file 1 [file mmc1.pdf]

iScience, Volume *29*

Supplemental information

Frozen parental care behavior in a pseudoscorpion trapped in Eocene Baltic amber

Chen Feng, Corentin Jouault, Qingqing Zhang, Arnoud Boom, Jana Christophoryová, Xiangbo Guo, Yuhe Gao, Chunpeng Xu, and Yu Liu

SUPPLEMENTARY TEXT AND FIGURE

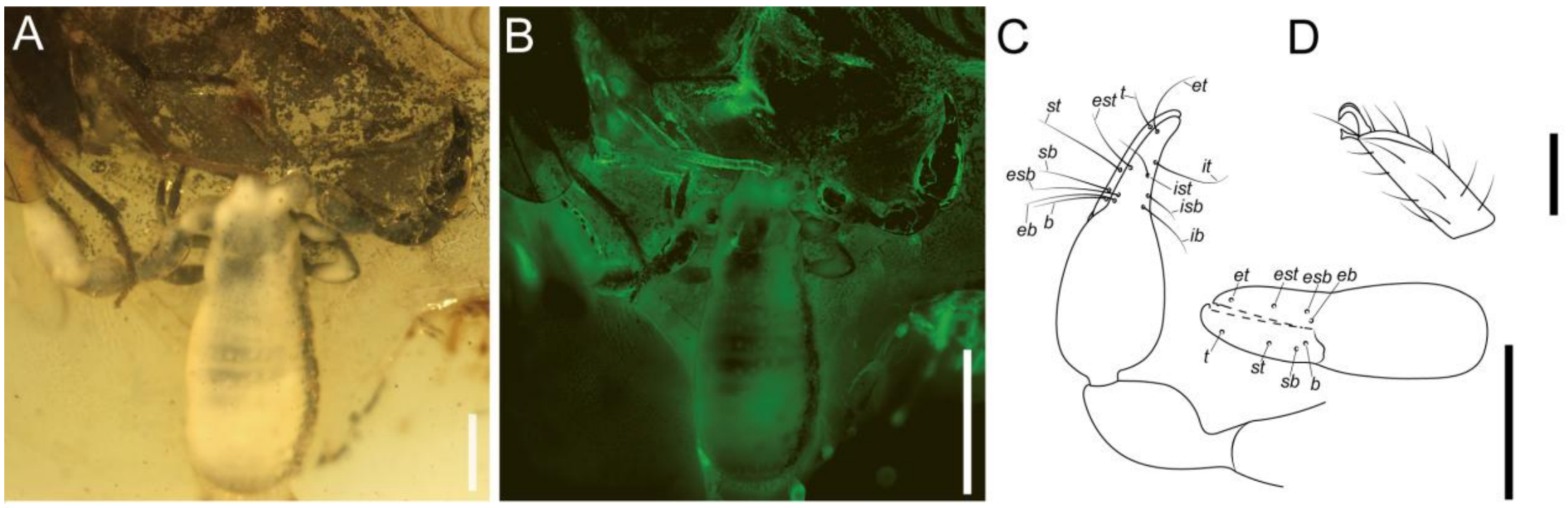

Supplementary Figure 1. Association of an iocheiratan pseudoscorpion with a dipteran host in Baltic amber. (A) Dorsal view of the iocheiratan female. (B) Wide-field fluorescence micrograph of the iocheiratan female. (C) Interpretative line drawing of the right pedipalp showing trichobothrial pattern. Abbreviations for the trichobothria: *b*, basal; *sb*, sub-basal; *st*, subterminal; *t*, terminal; *ib*, interior basal; *isb*, interior sub-basal; *ist*, interior subterminal; *it*, interior terminal; *eb*, exterior basal; *esb*, exterior sub-basal; *est*, exterior subterminal; and *et*, exterior terminal. (D) Interpretative line drawing of tarsus IV. Scale bars = 1 mm (A), 0.5 mm (B), 0.1 mm (C).

Systematic paleontology

Order Pseudoscorpiones de Geer, 1778

Group Homosphyronida Chamberlin, 1929

Remark. The new fossil possesses the diagnostic characters of Homosphyronida: trichobothrium *ib* and *isb* located on the lateral face of the chelal fingers, and metatarsi and tarsi of all legs fused, with those of the anterior legs having the same state as the posterior pair (Benavides et al., 2019).

Suborder Iocheirata Harvey, 1992

Remarks. Members of Iocheirata are characterised by the presence of venom glands in one or both of the chelal fingers (Harvey, 1992; Benavides et al., 2019). Previous studies also indicated that the absence of trichobothrium *xs* (Harvey, 1992) and the presence of the posterior maxillary lyrifissure (Benavides et al., 2019), together with the presence of venom glands are diagnostic of this group. In the new fossil, at least one venom gland is clearly visible, and trichobothrium *xs* appears to be

absent; however, the lyrifissure is difficult to discern.

Specimen 04-89

Remarks. Further systematic discussion is not possible because a white coating covering the specimen precludes detailed optical examination, particularly obscuring key diagnostic characters required for confident clade-level identification. In addition, the presence of pyrite within the amber, in the vicinity of the chelicerae, interferes with the acquisition of accurate 3D data by introducing artefacts during scanning. Consequently, no definitive taxonomic assignment is proposed, and the systematic placement of the fossil is not discussed in detail, as such interpretations would not be sufficiently supported by observable apomorphic characters.

Material. YKLP-AMB-04-89 ♀, a complete and well-preserved female specimen embedded in a piece of amber containing a brachyceran fly, housed in the amber collection of the Yunnan Key Laboratory for Palaeobiology, Institute of Palaeontology, Yunnan University, Kunming, China.

Locality and horizon. Baltic amber, Kaliningrad Oblast, Russia; late Eocene (likely Priabonian, ca. 34–38 Ma).

Description. Pedipalps and legs dark brown; body length ca. 1.98 mm (measured from the tip of the chelicerae to the abdominal apex); integument densely setose. Carapace slightly longer than wide, with a distinct transverse median furrow; chelicerae not observable owing to preservation. Abdomen with numerous setae distally. Pedipalp short and stout; patella almost spherical. Trichobothrial pattern as follows: on fixed finger, *eb* at base of finger; *est* at mid-level, and *esb* slightly closer to *eb* than to *est*; *ist* level with or slightly distal to *est*. On movable finger, *t* closer to *st* than to fingertip; *st* closer to *sb* than to *t*; *sb* nearer to *b* than to *st* (Supplementary Figure 1B). Female genital area with visible egg-sac. Pedal claw tips acute and curved; claw pads shorter than claws; sub-apical setae on leg IV simple.

Dimensions (in mm, ratios between parentheses): Pedipalp trochanter (not including protuberance) 0.26/0.17 (1.53×), femur 0.39/0.22 (1.77×), patella 0.43/0.22 (1.95×), chela (with pedicel) 0.85/0.32 (2.66×), chela hand (with pedicel) 0.53/0.32

(1.66×), movable finger 0.32/0.14 (2.29×). Carapace 0.59/0.51 (1.16×). Leg I trochanter 0.1/0.06 (1.67×), femur 0.14/0.08 (1.75×), patella 0.17/0.09 (1.89×), tibia 0.17/0.06 (2.83×), tarsus 0.19/0.04 (4.75×). Leg IV trochanter 0.14/0.12 (1.17×), femur+patella 0.32/0.13 (2.46×), tibia 0.27/0.07 (3.85×), tarsus 0.40/0.06 (6.67).

REFERENCES

Harvey, M.S. (1992). The phylogeny and classification of the Pseudoscorpionida (Chelicerata: Arachnida). Invertebr. Taxon. *6*, 1373–1408.
